# Temporal trend of syphilis in pregnancy and congenital syphilis by race

**DOI:** 10.1016/j.clinsp.2026.101024

**Published:** 2026-07-03

**Authors:** Rejane Calixto Gonçalves, André Calixto Gonçalves, Beatriz Mota Gomes, Aloisio Machado da Silva Filho, Olinda Carmo Luiz

**Affiliations:** a Department of Preventive Medicine, Faculdade de Medicina da Universidade de São Paulo (FMUSP), São Paulo, SP, Brazil; b CECS ‒ Center for Engineering, Modeling and Applied Social Sciences, Universidade Federal do ABC, São Bernardo do Campo, SP, Brazil; c Instituto de Saúde Pública, Universidade Federal da Bahia, Salvador, BA, Brasil

**Keywords:** Syphilis, Congenital syphilis, Pregnancy complications, Infectious, Health inequities, Ethnic and racial disparities in health

## Abstract

**Objective:**

to analyze the temporal trend in the incidence rate of syphilis during pregnancy and congenital syphilis in São Paulo between 2012 and 2021, according to race. A key focus of this analysis is to understand and highlight any racial differences in the incidence of syphilis.

**Methodology:**

This ecological study used data from the Notifiable Diseases Information System (SINAN) and the Live Births Information System (SINASC). The incidence rate was calculated by dividing the number of cases by the number of live births. The independent variables were 2012 to 2021 and race (White, Black, East Asian, Indigenous). Trends were analyzed using linear regression with the Prais-Winsten correction at the 5% significance level.

**Results:**

The findings reveal an increase in syphilis incidence during pregnancy and in congenital syphilis in São Paulo, with a disproportionate impact on the Black population. Among the White pregnant population, the Annual Percentage Change (APC) was 17.84% (p < 0.005), while among the Black pregnant population, it was 16.86% (p < 0.005). For congenital syphilis, the incidence among White newborns rose with an APC of 7.6% (p < 0.005), while in the Black population it rose by 6.17% (p < 0.005).

**Conclusion:**

In 2021, the number of cases among the Black population was 1.9-times higher than among the White population, underscoring persistent racial disparities and reinforcing the urgent need for equity-driven policies to prevent vertical transmission and control syphilis.

## Highlights

• The incidence of syphilis during pregnancy is increasing in São Paulo.
• Congenital syphilis rates are also rising in São Paulo.
• The Black population experiences a higher incidence of syphilis.
• Socioeconomic inequities have limited access to quality healthcare.
• Addressing racial disparities is crucial for eliminating congenital syphilis.

## Introduction

Syphilis is a systemic, chronic, curable bacterial infection that is exclusive to humans. When left untreated, it progresses through stages of varying severity, affecting multiple organs and bodily systems. It has been known for centuries; its etiological agent is *Treponema pallidum.* Its transmission occurs through sexual contact; however, the infection can be transmitted vertically to the fetus during the pregnancy of a woman with untreated or inadequately treated syphilis.^1^

Most people with syphilis have an asymptomatic form, which often goes unnoticed and can lead to the transmission of the infection to their sexual partners. However, it's crucial to remember that syphilis is a preventable disease. Timely and adequate treatment can significantly reduce the progression to more severe forms, especially during pregnancy, thereby preventing severe consequences such as miscarriage, prematurity, stillbirth, early or late congenital manifestations, and death of the newborn (NB).^1^

Congenital syphilis can be considered an indicator of the quality of prenatal care. Research published in 2019 highlighted the relationship between the lack of prenatal care and high rates of stillbirths caused by congenital syphilis. The study also highlighted the correlation between congenital syphilis rates and rates of infant mortality, spontaneous abortion, and stillbirth, reflecting the precarious conditions of maternal and child health in Brazil.^2^

The benzylpenicillin treatment is practical, low-cost, and accessible in the Brazilian Public Health System. Many untreated or inadequately treated cases can be successfully managed with the right interventions.

The scientific literature highlights the impact of prenatal care on women's lives and health, demonstrating that care is closely tied to sociodemographic, cultural, and economic factors that influence access to healthcare services. These studies also highlight that in societies characterized by inequalities, gender and racism constitute structuring elements and explain the unequal access of the Black women population to health services.^3^^,^^4^

A recent study revealed that maternal and congenital syphilis remain endemic in the country. A total of 15 million birth records were evaluated in Brazil between 2012 and 2017. The results indicated that brown women and the Black women population tend not to receive a timely diagnosis and adequate treatment for maternal syphilis, and their partners are less likely to be treated.^5^ This underscores the urgent need for comprehensive and targeted public health policies to address these racial disparities. *(5)*

In the municipality of São Paulo, since 2011, acquired syphilis has increased almost threefold annually, from 6712 to 18,645 cases in 2021.^6^

The primary objective of this study is to analyze the temporal trend of the incidence of syphilis in pregnancy and congenital syphilis in the city of São Paulo over a decade, with a specific focus on race and color.

## Materials and methods

This manuscript was prepared in accordance with the STROBE (Strengthening the Reporting of Observational Studies in Epidemiology) guidelines for observational studies.

The study population consisted of pregnant women and newborns residing in São Paulo.

The financial, corporate, and mercantile center of South America, the city is among the most populous in the world, with approximately 12.33 million inhabitants in 2021.^7^

The public Brazilian health system, Sistema Único de Saúde (SUS), serves the city of São Paulo, providing universal access to healthcare for the entire resident population and implementing specific strategies to control syphilis. With 469 primary healthcare units, the Municipal Health Department has adopted the Protocol for Preventing Vertical Transmission of Syphilis. The document outlines the actions required to control the disease, particularly in the care of pregnant women. Every pregnant woman should perform a rapid test for syphilis at the time of pregnancy diagnosis. Perform serology for syphilis in the first and second trimesters, in the 28th week of pregnancy, and the rapid test in the 32nd week, and perform the other routine prenatal exams.^8^ The pregnant woman's sexual partners should be treated and monitored to prevent reinfection, and all cases must be reported and monitored.

### Study design

Temporal trend of congenital syphilis and syphilis in pregnancy according to race/color, analyzed from an ecological design.

### Data sources

The authors relied on the National Information System on Notifiable Diseases (SINAN) and the Information System on Live Births (SINASC) as primary data sources.

### Outcome variables

All notifications of cases of congenital syphilis and syphilis during pregnancy of residents of the city of São Paulo (numerator), as well as all live births between 2012 and 2022 (denominator), were considered.

The incidence rate of congenital syphilis was calculated by dividing the number of reported cases by the number of live births.

The syphilis incidence rate in pregnancy was calculated by the ratio of the number of reported cases of syphilis in pregnancy divided by the number of live births.

To define a case of congenital syphilis, any newborn born to a woman with untreated syphilis or with treatment started at least 30-days before delivery was considered. Newborns with at least one of the following situations were also considered: clinical, cerebrospinal fluid or radiological manifestation of congenital syphilis and reactive nontreponemal test; Nontreponemal test titers of the infant higher than those of the mother, in at least two dilutions of peripheral blood samples, collected simultaneously at the time of delivery; ascending nontreponemal test titers in at least two dilutions in the follow-up of the exposed child; and; Titers of non-treponemal tests still reactive after six months of age, in a child adequately treated in the neonatal period; microbiological evidence of *Treponema pallidum* infection in a nasal discharge sample or skin lesion, biopsy.^9^

A case of syphilis was considered in a pregnant woman who, during prenatal care, childbirth, and the puerperium, presented at least one treponemal or non-treponemal reagent test, with any titration, with no record of previous treatment.^9^

### Exposures (independent variables)

Years 2012 to 2021.

Race (White, Black, East Asian, Indigenous). The authors introduced the term “East Asian” to refer to people of this origin and their descendants, primarily from Japan, China, and Korea. The Brazilian Institute of Geography and Statistics (IBGE) classification uses the term “Yellow” to refer to this ethnic group.

### Statistical analysis

The temporal trend of the rates was analyzed using Annual Percentage Changes (APC) according to race, with the respective 95% confidence intervals, using a linear regression model with a Prais-Winsten correction.^10^ All analyses were performed using R version 4 × (R Foundation for Statistical Computing, Vienna, Austria).^11^ The rates were considered ascending when the APC was upbeat and descending when negative. The rates were considered stable when the APC was not significantly different from zero (p-value > 0.05). To verify the presence of additional serial autocorrelation in the residuals, the Durbin-Watson test was utilized.

As a sensitivity analysis, the authors estimated linear regression models with a Prais-Winsten correction for first-order autocorrelation on the log-transformed rates for 2012–2021, including an indicator variable for race/skin color (Black or Brown vs. White) and temporal controls.

In the first specification, the authors adjusted for a linear time trend (log(rate) ∼ Black + year).

In the second, the authors introduced year fixed effects (log(rate) ∼ Black + i(year)), which absorb annual municipality-wide shocks ‒ such as changes in socioeconomic conditions, health policies, or macroeconomic fluctuations ‒ and therefore act as a proxy for area-level Socioeconomic Status (SES) that varies over time.

This approach enables the assessment of whether racial disparities persist after accounting for temporal variations in the municipality’s socioeconomic context.

## Results

Table 1 shows the period 2012 to 2021 annual growth trend in the number of cases and incidence rate of congenital syphilis in the White and Black populations. However, the results are higher in the Black population: incidence rose from 309 cases/thousand live births in 2012 to 472 in 2021, and the incidence rate increased from 4.6 in 2012 to 8.0 in 2021, compared with the White population. Whites had 280 cases per thousand live births in 2012, rising to 325 in 2021, with incidence rates increasing from 2.8 in 2012 to 5.8 in 2021. It is worth highlighting the magnitude of the variation: in the White population, the incidence rate increased by 48%, while in the Black population, it increased by 57.5% during the same period.

**Table 1:** Number of cases and incidence rate of congenital syphilis per thousand live births according to race/color population, municipality of São Paulo, Brazil, 2012 to 2021.

Year	White		Black		East Asian		Indigenous		Total	
	N	Rate	N	Rate	N	Rate	N	Rate	N*	Rate
2012	280	2.8	309	4.6	2	0.9	5	6.8	758	4.1
2013	304	3.4	385	5.8	7	2.3	3	2.1	911	5.1
2014	372	4.0	436	6.4	4	2.5	2	4.1	1045	5.6
2015	338	4.0	485	6.4	3	1.4	2	2.4	1077	5.8
2016	434	5.0	480	7.0	2	0.9	6	13.6	1165	6.6
2017	421	4.9	478	6.7	1	1.8	4	9.0	1154	6.5
2018	400	5.4	543	7.6	2	0.9	-	-	1263	7.2
2019	366	5.0	556	7.9	2	0.5	2	11.0	1241	7.3
2020	341	5.1	513	8.2	2	1.3	-	-	1154	7.4
2021	325	5.8	472	8.0	2	1.3	1	2.3	1021	7.3

Source: SINASC – National Live Birth Information System SINAN – National Information System for Notifiable Diseases.

⁎ Sum includes White, Black, East Asian, Indigenous, and other races. Some individuals did not respond.

Table 2 shows an annual growth trend in the White and Black pregnant women population. Still, it is noteworthy that in 2013, the number of cases in the Black pregnant women population was 1.5 times higher when compared to the White pregnant women population. In 2021, the number of instances of syphilis in the Black pregnant women population was 1.9-times higher, indicating an increase in racial inequality in the incidence of syphilis.

**Table 2:** . Number of cases and incidence rate of syphilis in pregnancy per thousand live births according to race/color population, municipality of São Paulo, Brazil, 2012 to 2021.

Year	White		Black		East Asian		Indigenous		Total	
	N	Rate	N	Rate	N	Rate	N	Rate	N*	Rate
2012	724	7.7	944	11.7	22	9.5	25	24.4	1778	10.0
2013	890	9.8	1368	17.0	24	11.1	13	12.6	2315	13.3
2014	994	10.9	1657	20.0	19	8.0	9	10.4	2693	15.2
2015	1074	12.0	1662	19.5	21	10.0	15	18.0	2839	16.0
2016	1211	14.4	2075	25.3	22	10.4	9	36.7	3406	20.2
2017	1510	18.3	2447	28.7	28	12.9	11	19.9	4129	24.2
2018	1806	22.8	2861	33.5	48	22.9	11	26.4	4902	29.3
2019	1849	24.8	3127	37.9	37	20.6	14	51.6	5235	32.8
2020	2064	30.3	3826	49.1	55	35.3	8	30.5	6109	41.3
2021	1930	32.4	3711	53.2	38	31.5	6	29.7	5749	43.8

Source: SINASC – National Live Birth Information System SINAN – National Information System for Notifiable Diseases.

⁎ Sum includes White, Black, East Asian, Indigenous, and other races. Some individuals did not respond.

**Fig. 1:**
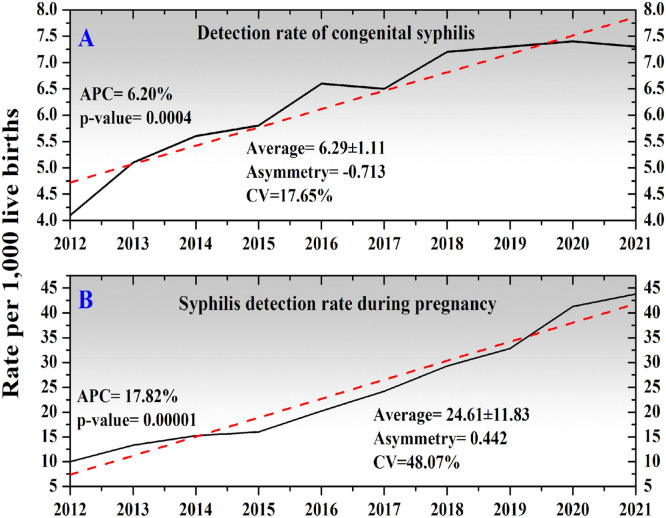
Incidence of congenital syphilis and in pregnancy in the city of São Paulo, 2012 to 2021. SINASC – National Live Birth Information System SINAN – National Information System for Notifiable Diseases.

The incidence rate in the White pregnant women population went from 7.7 in 2012 to 32.4 in 2021, an increase of 4.2-times in the period, and for the Black pregnant women population, it went from 11.7 in 2012 to 53.2 in 2021, an increase of 4.55-times (Fig. 1).

The incidence of Congenital Syphilis (CS) showed a significant upward trend (p-less than 0.0004) in the period evaluated, with an APC of 6.20%. However, CS incidence stabilized from 2018 onward, with a slight decline.

The rate of syphilis detection in pregnant women showed an upward trend in the same period, from 10.0 cases/thousand live births in 2012 to 43.0 cases/thousand live births in 2021, with an APC of 17.82%. In 2014, the syphilis incidence in pregnant women was 15.0 cases/thousand live births, with stability in 2015 and a progressive increase until 2019 (32 cases/thousand live births), 42 cases/thousand live births (2020), and 43 cases/thousand live births (2021).

**Fig. 2:**
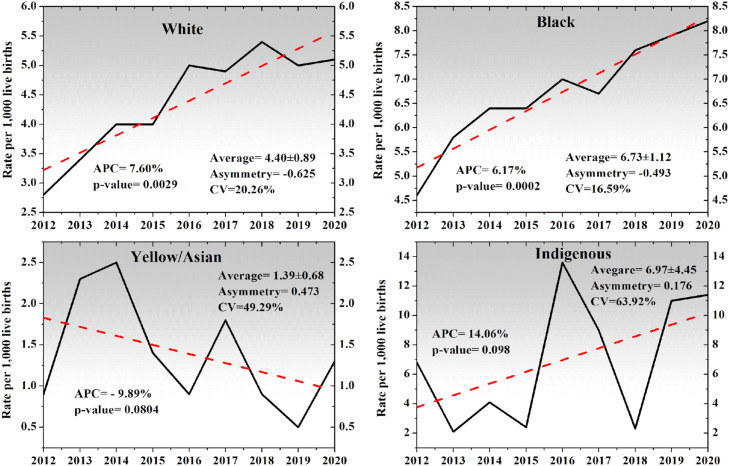
Incidence of congenital syphilis, according to race and color, in the city of São Paulo, 2012 to 2020. SINASC – National Live Birth Information System SINAN – National Information System for Notifiable Diseases.

The analysis of the incidence of congenital syphilis (Fig. 2) shows that the incidence of congenital syphilis in the White population shows an upward trend, with an APC of 7.6% and p less than 0.005.

**Fig. 3:**
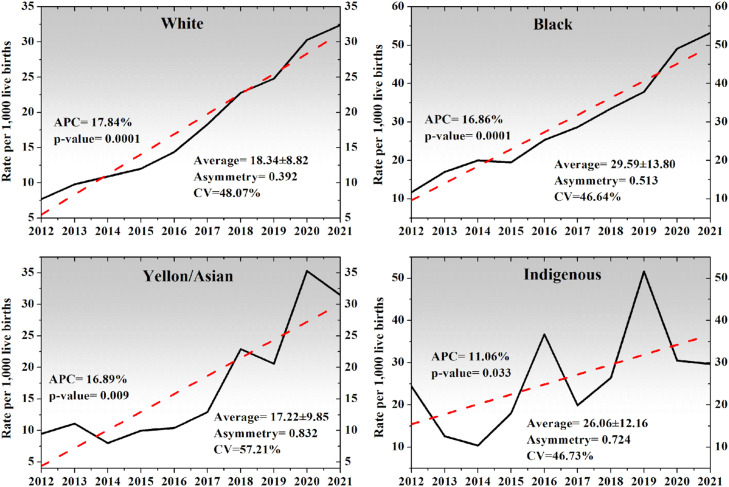
Incidence in pregnant women, according to race and color, in São Paulo, 2012 to 2021. SINASC – National Live Birth Information System SINAN – National Information System for Notifiable Diseases.

When the authors analyzed the incidence of congenital syphilis in the Black population in the same period, they also found an upward trend with an APC of 6.17 and p < 0.005. When the authors compare the behavior of the incidence of congenital syphilis in the White and Black populations, they observed that although both populations show an upward trend, the rates are always higher in the Black population (Fig. 3).

The syphilis incidence in pregnant women showed a significant upward trend in the population of the White pregnant women population, with an APC of 17.84% and p-equal to 0.0001. During the same period, the APC was 16.86% of the Black population, with a p-value of 0.0001. In this case, the Black population also has a higher incidence of syphilis in pregnant women when compared to the White pregnant population.

For Indigenous and East Asian populations, interpreting temporal trends is limited by the small number of cases and births reported annually. Both groups exhibited wide year-to-year fluctuations, reflected in high coefficients of variation (63.9% for Indigenous) and non-significant APC estimates (p > 0.05). These findings indicate statistical instability rather than consistent epidemiological change. The low counts are likely influenced by underreporting and misclassification of race/color in health information systems, especially for populations with smaller denominators. Therefore, while the inclusion of these groups is for transparency and equity monitoring, their trends should be interpreted with caution, as they do not provide robust evidence of changes in incidence over time.

The sensitivity analysis showed that in models including year fixed effects, the rate ratio (black or brown vs. white) remained 1.52 (95% CI 1.43–1.63) for congenital syphilis and 1.63 (95% CI 1.55–1.70) for syphilis in pregnancy, indicating that racial disparities persisted even after adjusting for time-varying area-level socioeconomic conditions.

## Discussion

The incidence of syphilis in pregnancy and congenital syphilis showed an upward trend in the city of São Paulo in the period 2012 to 2021. Although the incidences of syphilis in pregnant and congenital syphilis increased in both the White and Black populations, these results were higher in the Black women population.

Between 2020 and 2021, the incidence of congenital syphilis remained stable or slightly decreased compared to 2019, while syphilis in pregnancy continued to increase during the same period.

The primary models ‒ without adjustment for socioeconomic variables ‒ reflect the total burden of racial inequality, encompassing both socioeconomic and potential institutional factors. The sensitivity analysis demonstrates that racial disparities remained robust even after accounting for time-dependent socioeconomic variation.

According to Lessa et al. (2022), the Black women population is less likely to start prenatal care before 12-weeks of pregnancy, have six or more consultations, undergo HIV tests and VDRL exams, and receive guidance regarding care during pregnancy and childbirth. The authors state that being a Black woman in Brazil and occupying unfavorable social positions entails disadvantages for women in terms of access to adequate prenatal care.^12^^,^^13^

Morais et al. (2019) state that Black women have recurrent health demands. Socioeconomic data show that in Brazil, most Black women live below the poverty line, corroborating the thesis that syphilis, as well as other STDs, are preeminent in specific risk groups, that suggests a need to implement preventive and care public policies that value democratization and equity in healthcare.^14^ which considers not only the prompt and appropriate treatment of pregnant women and their sexual partners, but also the importance of active surveillance and training for health professionals. This training should focus on both the diagnosis and management of syphilis. Additionally, it calls for the relevant authorities to adopt effective measures to reduce racial inequity.

Racial and ethnic disparities in syphilis rates among pregnant women and in vertical transmission continue to reveal inequities in the control of Sexually Transmitted Infections (STIs). Across countries with varying income levels, some progress has been made in equity-focused strategies; however, the outcomes remain insufficient to bridge existing gaps.

In the United States, the recent federal response prioritizes reducing congenital syphilis with a strong emphasis on racial and ethnic equity. The American College of Obstetricians and Gynecologists (ACOG) now recommends screening all pregnant women for syphilis at the first prenatal visit, again in the third trimester, and at delivery in settings of high incidence or vulnerability.^15^ Partner notification efforts have also been intensified, along with initiatives addressing structural barriers, such as health insurance coverage, access to medication (benzathine penicillin), and coordination with social services. These measures indicate a broader approach than testing and treatment alone. Nevertheless, as recent reviews have shown, despite updated guidelines, the burden of syphilis remains disproportionately higher among Black, Hispanic, and Indigenous women, demonstrating that universal interventions are not sufficient without explicit equity-focused measures.^16^

In the United Kingdom, the national syphilis action plan and recent parliamentary reports highlight targeted screening, partner notification, and awareness campaigns focused on disproportionately affected populations, alongside the use of priority-setting frameworks to reduce inequalities in sexual health services. For instance, data from the Integrated Screening Outcomes Surveillance Service (ISOSS) reported that, in 2023, 41% of heterosexual syphilis diagnoses occurred among women living in the most socioeconomically deprived quintile, and that infection rates were relatively high among Black and minority ethnic women. However, most cases still occurred among the White women population.^17^ These findings underscore that even in high-income settings, social vulnerability remains closely linked to the risk of infection.

In the Americas, the Pan American Health Organization (PAHO/WHO) launched the Elimination of Mother-to-Child Transmission of STIs initiative, which incorporates equity indicators such as screening and treatment coverage among pregnant women by racial or ethnic subgroup, and validation of results by race/ethnicity or region.^18^ Such disaggregated monitoring enables countries to identify high-risk groups and implement targeted interventions.

In Brazil, national epidemiological bulletins show persistent racial and ethnic disparities: one national study estimated that among mixed-race women, 46% of maternal syphilis and 52% of congenital syphilis cases were preventable. In contrast, among the Black women population, these proportions were even higher.^19^ Another study found that among Indigenous women in Central Brazil, the detection rate of syphilis in pregnancy reached 35.2 per 1000 live births, and the incidence of congenital syphilis was 15.7 per 1000 live births ‒ approximately twice the national average. These findings underscore the need for universal policies to incorporate equity-oriented mechanisms and a clear focus on vulnerable populations.

Health systems play a pivotal role in ensuring equitable access to prenatal syphilis screening and treatment. Primary Health Care (PHC) serves as the leading platform for early detection and timely treatment. WHO and PAHO guidelines, as well as national protocols, recommend universal screening in the first trimester, retesting in the third trimester and/or at delivery in high-incidence settings, same-day testing and treatment, and continuous supply of benzathine penicillin in PHC facilities.

In summary, although significant progress has been made in different contexts ‒ from the United States and the United Kingdom to Latin America ‒ the systemic impact on racial and ethnic disparities remains limited.

### Limitations

The study was reported in accordance with the STROBE recommendations, which strengthens the transparency and completeness of the manuscript; however, as with any observational study, limitations remain.

A key limitation of this study is that congenital syphilis rates were calculated using live births as the denominator, in accordance with WHO, PAHO, and Brazilian Ministry of Health standards to ensure comparability across surveillance systems and over time.^15^, ^16^, ^17^ However, this approach inherently captures only the burden among live-born infants and does not reflect the total reproductive burden of infection, which also includes fetal and perinatal losses. Evidence from Brazil indicates that fetal deaths represent approximately 1–1.5 % of total births.^18^ and global estimates suggest that stillbirths linked to untreated maternal syphilis account for a substantial proportion of preventable losses.^5^ The exclusion of stillbirths likely results in systematic underestimation of the actual disease burden, and this bias may disproportionately affect Black women, among whom late diagnosis and inadequate prenatal care are more frequent.^19^, ^20^, ^21^, ^22^

While the health information system in the city of São Paulo is generally considered of reasonable quality, there may still be issues such as underreporting or overreporting of cases. To tackle underreporting and improve the quality of information systems, the City Hall of São Paulo has implemented several strategies. These strategies include ongoing training for health professionals, collaboration with hospitals and notary offices, and the use of technological tools. Furthermore, a strict protocol for mandatory notification requires that negative reports be submitted when no incidents occur.

Since this is an ecological study, there is a risk of committing fallacies when making inferences about individuals from aggregate data from groups or populations. This issue is common in ecological studies, where the units of analysis are groups rather than individuals. As a result, incorrect conclusions may arise if an association observed at the aggregate level also applies to individuals. However, the objective of this study is to identify racial differences within the population, not to use the results to inform individual decisions, but rather to provide insights that contribute to the development of health policies.

## Conclusions

Health policies should prioritize early and universal screening of pregnant women. This refers to conducting syphilis tests at the beginning of prenatal care and again in the third trimester. Prompt treatment with benzathine penicillin for pregnant women diagnosed with syphilis and their sexual partners is essential to prevent reinfection. Additionally, postnatal follow-up for newborns is crucial for diagnosing congenital syphilis and ensuring appropriate treatment if necessary.

To effectively manage this issue, the following measures are recommended:

1. Awareness Campaigns: Promote prevention strategies such as condom use, the importance of prenatal care and testing for syphilis, as well as reproductive rights and family planning.
2. Monitoring and Epidemiological Surveillance: Conduct studies to identify regions with high rates of congenital syphilis to prioritize targeted actions.
3. Integration of Services: Ensure collaboration between primary care, maternity hospitals, and urgent care services to guarantee continuity of care and follow-up for pregnant women and their infants.
4. Expansion of Care Access: Increase the coverage of primary care services and extend their operating hours, particularly in areas with higher syphilis occurrence, along with other measures to enhance access.
5. Hiring Qualified Professionals: Recruit enough qualified health professionals and provide ongoing training on the importance of syphilis prevention, diagnosis, and treatment, including discussions on racial disparities. Require comprehensive and regularly audited anti-bias training for prenatal healthcare professionals.
6. Support for Pregnant Women: Establish early and universal screening for pregnant women who do not receive prenatal care or who miss appointments. This can be done through home visits across the community. Additionally, provide prompt, accessible diagnoses of pregnancy to encourage earlier access to prenatal care.
7. Resource Management: Ensure the availability of administrative processes and resources for the continuous acquisition and distribution of supplies needed for diagnosis and treatment.
8. Establish and oversee standardized clinical protocols to reduce subjectivity in diagnosis and treatment.
9. Provide funding for culturally sensitive prenatal programs directed by Black healthcare professionals.
10. Develop effective systems to report and address instances of patient discrimination.

Eliminating congenital syphilis in São Paulo city will require not only universalization of screening and treatment but also explicit equity-focused policies, service integration, race-disaggregated monitoring, and strengthened primary and community health systems. Effective data-driven surveillance allows health systems to identify “critical points” ‒ for example, prenatal clinics with low screening coverage or a high proportion of unmonitored pregnancies ‒ and to allocate resources more equitably.

Finally, integrating sexually transmitted infection and reproductive health services with community-based interventions ‒ such as outreach testing, digital partner notification, transport assistance, and social support ‒ is essential to overcome financial, geographic, and social barriers that exacerbate racial and ethnic inequities.

## Author’s contribution

RCG: Conceptualization; methodology; writing; original draft preparation; and data curation.

ACG: Conceptualization; visualization; investigation.

BMG: Conceptualization; visualization; investigation.

AMSF: Conceptualization; methodology; software; validation.

OCL: Conceptualization; methodology; supervision.

## Ethical aspects

The study was approved by the Research Ethics Committees of FMUSP (Opinion n°6,846,974) and the Municipal Health Department of São Paulo (Opinion n° 6900,005). As it was based on publicly available secondary data without individual identifiers, informed consent was not required, in accordance with CNS Resolution n°510/2016.

## Funding

CAPES has awarded a Master's scholarship to Rejane Calixto Gonçalves in the Postgraduate Program in Public Health at FMUSP.

## Declaration of competing interest

All authors reviewed and approved the content, declaring no conflicts of interest.

## Data Availability

The datasets generated and/or analyzed during the current study are available from the corresponding author upon reasonable request.
